# Challenges and caveats in manipulating extracellular vesicle secretion from pancreatic cancer cells

**DOI:** 10.1080/15384047.2025.2569946

**Published:** 2025-10-25

**Authors:** Jennifer M. Finan, Jonathan R. Brody

**Affiliations:** a Department of Surgery, School of Medicine, Oregon Health & Science University, Portland, OR, USA; b Department of Cell, Developmental and Cancer Biology, Oregon Health & Science University, Portland, OR, USA; c Brenden-Colson Center for Pancreatic Care, School of Medicine, Oregon Health & Science University, Portland, OR, USA; d Knight Cancer Institute, Oregon Health & Science University, Portland, OR, USA

**Keywords:** Pancreatic cancer, extracellular vesicles, rab GTPase

## Abstract

**Background:**

Extracellular vesicle (EV) signaling is important in multiple malignancies, including pancreatic ductal adenocarcinoma (PDAC). In this coordinated cell‒cell signaling mechanism, genetically altered tumor cells signal to surrounding normal cells to promote tumor progression. Many efforts have been made to mechanistically interrogate this signaling axis by inhibiting EV secretion from cells. These techniques leverage our understanding of how EV biogenesis interferes with ceramide production or GTPase activity, which aids in membrane fusion with the plasma membrane.

**Material and methods:**

Our group leveraged these methods in our orthotopic PDAC mouse model to investigate the importance of PDAC EV secretion. We interfered with the GTPases Rab27a and Rab35 and utilized an inhibitor of ceramide production (GW4869) to ablate EV secretion.

**Results and conclusion:**

Overall, we found that these models did not perform as anticipated, and we could not consistently inhibit KPC cell EV secretion. These results emphasize the challenges of interfering with EV secretion, as several parallel pathways, such as direct membrane budding, can compensate. Further studies are needed to develop models for studying the role of EVs *in vivo*.

## Introduction

Extracellular vesicle (EV) cell–cell signaling has been established to promote tumorigenesis in multiple cancers, including pancreatic ductal adenocarcinoma (PDAC).^1-5^ Inhibiting EV release and thus signaling has been an appealing avenue for understanding the mechanisms of EV signaling and their role in shaping both paracrine and endocrine signaling within organisms. EVs are produced through multiple biogenesis pathways, all of which rely on specific proteins to enable membrane fusion or budding from the plasma membrane.^6^ Thus, to inhibit EV secretion, many have interfered with these proteins to ablate EV secretion both *in vitro* and *in vivo*. However, multiple technical limitations exist, such as protein redundancy and compensatory mechanisms for EV release through alternative biogenesis pathways. Thus, we aimed to evaluate the current techniques to inhibit EV secretion and utilize these models to characterize the importance of PDAC EV signaling.

Three main approaches have been utilized in cancer models to decrease EV signaling: (1) targeting the ESCRT machinery, (2) disrupting Rab GTPases, and (3) small molecule inhibition of sphingolipid metabolism.^7^^,^^8^ In PDAC, Dr. Sonia Melo and colleagues previously reported that interfering with Rab27a decreased EV secretion from both human and mouse PDAC cells.^4^ They reported reduced tumor growth in an immunocompromised mouse model with tetracycline-inducible knockdown (KD) of Rab27a.^5^ Further, they showed that tumor progression decreased in KPC mice with small molecule inhibition of EV release from all cells leveraging Rab27a inhibitor Nexinhib20.^4^ Dr. Yuliya Pylayeva-Gupta and colleagues further investigated the role of Rab27a in PDAC progression and metastasis.^9^ They found that Rab27a knockout (KO) increased epithelial-to-mesenchymal transition (EMT), increasing cell invasiveness. Interestingly, this phenotype was also attributed to decreased metastatic seeding, which the authors claim may be due to the inability of these cells to undergo mesenchymal-to-epithelial transition (MET), restricting metastatic outgrowth.

Some groups have aimed at optimizing the currently available small-molecule inhibitors of EV secretion. For example, a panel of compounds was tested in triple-negative breast cancer cell lines. They found that even with inhibitors that reduce EV release by 98%, there were still significant phenotypic impacts of the remaining 2% of EVs, suggesting that inhibiting EV secretion is likely quite challenging due to the lipid dynamics and multiple pathways through which cells can release EVs.^10^

Our laboratory employed previously published tools that interfere with Rab27a, Rab35, or ceramide production in mouse PDAC cells to determine the impact of ablating EV secretion in our immunocompetent orthotopic mouse model.^4^^,^^5^^,^^8^^,^^11^ Through this work, we found that these models did not significantly decrease EV secretion reliably, and we found no changes in tumor growth *in vivo*. These findings highlight the need for improved strategies to study the role of EV signaling *in vivo*. Further, these findings highlight potential pitfalls in previous reports of ablating EV secretion *in vivo*, where no such validation on tumor interstitial fluid to ensure adequate EV knockdown.

## Materials and methods

### Cell lines

A mouse PDAC cell line (KPC-8069) was gifted by Dr. Michael A. (Tony) Hollingsworth (University of Nebraska Medical Center) and cultured in DMEM supplemented with 10% fetal bovine serum (FBS) and 1% penicillin‒streptomycin. The cell lines were cultured at 5% CO2 at 37 °C in a humidified atmosphere and routinely tested for *Mycoplasma*. Novogene performed CRISPR/Cas9-mediated genetic deletion of Rab27a and Rab35 with guide RNAs: CUUGGCCUUGGGAGACUCUG (Rab27a) and UCGGACUGUGGAGAUCAACG (Rab35).

### Extracellular vesicle isolation

As previously published, KPC-8069 cells were plated at 2 × 10^6^ per 15 cm dish.^12^ The next day, the media was changed to DMEM supplemented with 10% EV-depleted FBS, which was generated by filtering FBS with an Amicon stirred cell (EMD Millipore UFSC40001) using a 300-kDa ultrafiltration disk (EMD Millipore PBMK07610). After 48 h, the conditioned media was collected and spun at 2000 × *g* for 10 min to remove cell debris. The media was concentrated utilizing Amicon Ultra-15 Centrifugal Filter Unit (Millipore #UFC9100) filter columns down to 1 mL. Size exclusion chromatography (SEC) was performed utilizing Izon qEV1 columns with 0.22 µm-filtered PBS, and EVs were collected from pooled fractions 7–10. Simultaneously, the cells were trypsinized following conditioned media collection for cell counting and pelleting for EV particle concentration normalization and controls via immunoblotting.

### Immunoblotting

The cell pellets were washed in PBS and lysed using RIPA lysis buffer (Thermo Scientific #89900) with Halt™ Protease Inhibitor Cocktail (Thermo Scientific #87786). For immunoblotting, the protein concentration was quantified utilizing Pierce™ BCA Protein Assay Kit (Thermo Fisher, #23225), and 20 µg samples were prepared in 25 µl of 5× loading buffer. SEC fractions were prepared by suspending 20 μL of SEC sample in 5 μL of 5× loading buffer. The samples were resolved using 10% SDS‒PAGE and transferred to a polyvinylidene difluoride membrane (Bio-Rad #1620264). The samples were resolved on 10% SDS‒PAGE and transferred to PVDF membranes (Bio-Rad, #1620264). The membranes were incubated with Ponceau, blocked (Li-Cor #927-5000), and incubated with primary antibodies diluted in blocking buffer overnight at 4 °C. The membranes were rinsed, incubated with secondary antibody (1:20,000), and visualized on an iBright™ FL 1500 Imaging System. Immunoblots were quantified utilizing ImageJ and normalized to the loading control.

### Transmission electron microscopy

The SEC fractions were submitted to the Oregon Health & Science University (OHSU) Multiscale Microscopy Core for sample processing and imaging. The samples were placed on 200-mesh grids coated with carbon and Formvar for 3 min. The grids were rinsed thrice in water and exposed to 1% (w/v) uranyl acetate for 3 min. The grids were blotted dry and imaged on a Thermo Fisher Scientific Tecnai transmission electron microscope operated at 120 kV and equipped with an AMT NanoSprint12 camera.

### Fluorescent nanoparticle tracking analysis

Lipid dye, Di-8-ANEPPS (Biotium #61012), was prepared by diluting 1:100 in PBS with 0.05% Pluronic F-127. 20 µL of each sample was incubated with 1 µl of dye for 15 min and immediately diluted with 979 µl of H_2_O. The samples were run on the ZetaView utilizing the 488 nm laser with 500 nm filter at 11 positions at 25 °C. The particle concentration was reported and normalized to the cell count of donor cells.

### Transfection and lentiviral transduction

Cell lines were developed to express the tetracycline-inducible shRab27a for mouse Rab27a (pLKO-Tet-On-shRab27a, Addgene #120930). Lentivirus was generated from LentiX HEK293T cells that were transfected utilizing LentiX™ Packaging Single Shots with 7 µg of transfer plasmid. Dr. Ferdinando Pucci generously gifted lentivirus for Rab35 S22N DN mutant (p27.pA.NGFR.mCMV.hPGK.Rab35(S22), Addgene #226766) and control (p25.pA.NGFR.mCMV.hPGK.Rab35(WT), Addgene #) and stored at −80 °C until transduction. The cells were then transduced with the appropriate lentiviral supernatant combined 1:1 with DMEM and polybrene. Following 24 h of transduction, the cells were selected with neomycin. The concentrations of the selection agents were determined as the concentration that killed WT cells after 4 d of treatment.

### DNA validation

The cells were pelleted, and the DNA was isolated using the Qiagen DNeasy kit. DNA was amplified using the following forward and reverse primers for shRab27a: GAAGCCATAGCACTCGCAGAGA and CAGGACTTGTCCACACACCGTT. DNA was separated on a 1.5% agarose gel to ensure the insertion of the entire construct.

### Reverse transcription‒quantitative polymerase chain reaction (RT‒qPCR)

RNA was isolated from cell pellets utilizing the Qiagen RNeasy (#74106) column purification with the addition of DNase treatment. The RNA concentration and quality were assessed utilizing a NanoDrop spectrophotometer. Complementary DNA (cDNA) was made from 1000 ng of RNA using the High-Capacity cDNA Reverse Transcriptase Kit (#4368813). Relative quantification was assessed using the 2−ΔΔCt method, and GAPDH was used for normalization. Melting curves of the qPCR product were evaluated to ensure amplification product specificity.

### Fluorescence-activated cell sorting (FACS)

Cells were suspended in FACS buffer (PBS with 1% BSA, 1 mM EDTA) and strained in polystyrene tubes. Single-cell clones were generated by FACS sorting one cell per well of a 96-well plate and expanding each clone prior to validation.

### LNGFR bead separation

The cells were harvested and suspended on ice for 15 min with MACSelect LNGFR MicroBeads (Miltenyi Biotec #130-091-330) per the manufacturer's instructions. They were then separated using magnetic separation per the manufacturer's instructions and compared to the flow through to ensure proper selection and purity.

### Cell growth assay

Cells were plated in a 96-well plate at 500 cells/well in 100 µL DMEM. The media was aspirated from one row of cells the following day and each subsequent day. The cells were incubated in 100 µL of H_2_O for 1 h, and the H_2_O was then transferred to a black 96-well plate and stored at −80 °C for the duration of the experiment. At the endpoint, the level of DNA was quantified utilizing Quanti-iT™ PicoGreen™ dsDNA Assay Kit per manufacturer's instructions.

### Mouse studies

All mouse protocols were outlined in the Institutional Animal Care and Use Committee (IACUC) protocol #00003322 and were approved by the OHSU Department of Comparative Medicine. Utilizing the established pancreatic orthotopic survival surgery, PDAC cells were injected directly into the tail of the pancreas of 9-week-old male C57BL6 mice from Jackson Laboratories, as the KPC-8069 cell line was derived from a male mouse. The cells were grown in culture and collected within the log growth phase, suspended in 1:1 cold PBS:Matrigel, 20 µl per injection. After injection, the needle was exchanged with a cotton swab to eliminate leakage of cells. The peritoneum was closed using absorbable Vicryl Rapide sutures (VR834, McKesson, Irving, TX), and the skin was closed using wound clips (#12022-09, Fine Science Tools, Foster City, CA). After surgery, the mice were injected with 0.1 mg/kg buprenorphine, provided wet food, and monitored daily for one week for any signs of stress. Seven days following surgery, the wound clips were removed. The mice were weighed weekly and euthanized after 14 d. The mice were anesthetized with isoflurane and euthanized with CO₂ in accordance with American Veterinary Medical Association (AVMA) guidelines and OHSU IACUC policies. To determine the impact of Rab27a or Rab35 KO in mice, we orthotopically implanted WT (scramble control guide RNA) KPC-8069, Rab27a KO, or Rab35 KO cells into the mice (*n* = 5 per group). To assess the impact of EV secretion inhibition in our mouse model, 15 mice were orthotopically implanted with WT KPC-8069 cells and randomized into groups and treated with vehicle (control), 2.5 mg/kg GW4869, or 5 mg/kg GW4869 intraperitoneally every day (*n* = 5 per group). The experimental unit was a single mouse. No formal a priori sample size calculation was performed; group sizes were based on prior similar studies. The mice were randomized using a random number generator. The mice were excluded from the study if there were leaks during orthotopic implantation (no exclusions occurred in this study). The investigator performing surgeries and treatments was aware of group allocation, but outcome assessments and data analysis were conducted blinded to group assignments. The mice were housed in ventilated cages on a 12-h light/dark cycle with ad libitum access to standard chow and water, and environmental enrichment was provided.

### GW4869 kill curve

The cells were plated in a 96-well plate at 500 cells/well in 100 µL DMEM. The following day, GW4869 was added at 2× in serial dilutions in 100 µL of DMEM. After 4 d, relative cell survival was quantified utilizing CellTiter-Glo® 2.0 Cell Viability Assay according to the manufacturer's instructions.

### Tumor flow cytometry

Tumors were collected and kept on ice in FBS-free DMEM. Tumors were then dissociated utilizing GentleMacs and the cells were strained through a 40 µm filter, spun at 200 × *g* for 5 min, and then incubated with ACK lysis (Gibco™ #A1049201) for 1 min to lyse all red blood cells. The cells were again spun for 5 min at 200 × *g* and blocked in Mouse BD Fc Block™ (BD #553142) diluted 1:100 with fixable LIVE/DEAD™ (Invitrogen #L23105) diluted 1:500 in PBS for 30 min at 4 °C. Following blocking, the cells were rinsed and resuspended in staining solution with conjugated antibodies diluted in PBS with 2% FBS and 0.5 mM EDTA for 1 h at 4 °C in the dark (CD45.2 PE Cy7 BioLegend #109830, 1:100). The cells were then rinsed twice, strained, resuspended in 200 µL of CountBright™ beads (Invitrogen™ #C36950), and run on a Cytek® Aurora 5-Laser Spectral Flow Cytometer. All experiments included single-color controls and fluorescence-minus-one controls for gating. Flow cytometry data were analyzed utilizing FlowJo. Events were gated on cell size, singlets, and live, followed by gating for immune (CD45^+^) and nonimmune (CD45^−^) cells. Populations of interest were gated, and cell numbers were calculated as percentages of GFP^+^. The fluorescence intensities were calculated from the geometric mean.

### Statistics

All the statistical analyses were performed using GraphPad Prism (version 10.1.2). *In vitro* data are presented in bar plots, with 3 representative replicates shown without single data points as the mean ± standard deviation. In contrast, *in vivo* data are presented with single datapoints as the mean ± standard error of the mean. All experiments were performed in biological replicates, defined as independent experiments conducted on distinct biological samples, either separate cell culture experiments or individual animals. Exact *n* values for biological replicates are reported throughout the figure legends. Statistical tests were unpaired two-tailed Student's *t*-test for comparing two groups and one-way ANOVA for multiple group comparisons.

### Ethics statement

All animal procedures were conducted in accordance with institutional guidelines and approved by the OHSU IACUC protocol #00003322.

## Results

**Figure 1.**
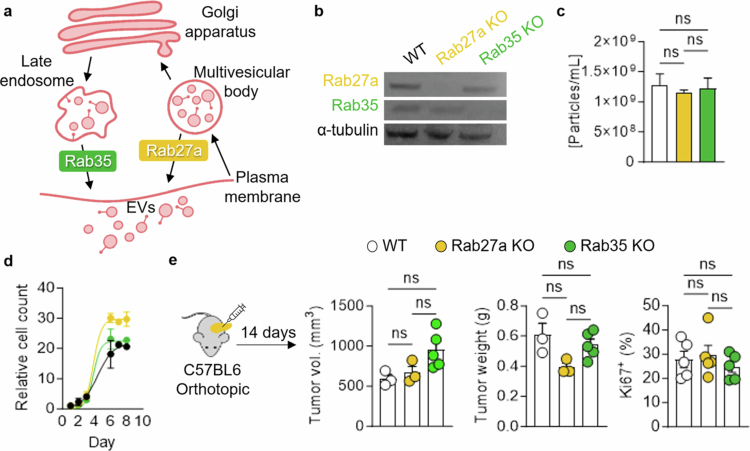
Rab27a and Rab35 KO do not impact KPC tumor growth. (a) Schematic of the involvement of Rab27a and Rab35 in EV secretion. (b) Immunoblot validation of Rab27a and Rab35 KO probed for Rab27a, Rab35, and α-tubulin. (c) Particle concentration of EVs derived from WT, Rab27a KO, and Rab35 KO cells. (d) Growth of WT, Rab27a KO, and Rab35 KO cells. (e) Tumor volume, weight, and immunofluorescence staining for Ki-67 in KPC WT, Rab27a KO, and Rab35 KO 14-d orthotopic pancreatic tumors implanted into C57BL6 mice. *p*-values were calculated using an ordinary one-way ANOVA. *p < 0.05; **p < 0.01; ***p < 0.001; ns, not significant.

We aimed to employ established techniques to suppress EV signaling *in vivo*. Among the most established techniques are genetic or small molecules that target Rab27a and Rab35, two crucial GTPases involved in membrane fusion events upstream of EV release (Figure 1a). To assess the role of Rab27a and Rab35 in our orthotopic mouse model of PDAC, we utilized CRISPR/Cas9 to genetically delete *Rab27a* or *Rab35* from PDAC cells derived from the *Kras*^*G12D*^*; Trp53*^*R172H*^*; Pdx1-Cre* (KPC) genetically engineered mouse model (Figure 1b). The pooled KO cell lines had a KO purity of 93% (Rab27a KO) and 94% (Rab35 KO) and were compared to scramble control guide RNA-targeted cells, referred to as WT cells, throughout this study. We isolated EVs from conditioned media from our KPC cells leveraging size exclusion chromatography (SEC) following MISEV guidelines.^13^ As previously published, we validated our EV isolation via protein content, immunoblotting for classical EV markers, nanoparticle tracking analysis, and transmission electron microscopy (Supplementary Figure 1S).^12^ Indeed, EVs were contained in SEC fractions 7−10 based on the protein content; the presence of the classical EV markers CD81, TSG101, and ALIX; and membranous particles observed via transmission electron microscopy. However, upon EV isolation from our Rab27a KO and Rab35 KO cells, we found no decrease in EV secretion compared to WT cells (Figure 1c). The KO of either GTPase did not impair *in vitro* KPC PDAC cell growth (Figure 1d). *In vitro* monoculture systems lack stromal interactions, immune components, and the extracellular matrix, all of which are known to impact EV secretion and uptake; thus, we reasoned that Rab27a- or Rab35-dependent EV secretion might only be revealed *in vivo* within the complex TME. Therefore, we orthotopically implanted WT, Rab27a KO, or Rab35 KO cells into the pancreas of immunocompetent mice and collected tumors after 14 d as previously established.^12^ Tumor growth was not impaired with Rab27a or Rab35 KO cells, and overall cell proliferation, as stained for by Ki-67 was unchanged (Figure 1e). Together, these data suggest that genetic KO of either of these GTPases may induce compensatory mechanisms for EV release in our model. An alternative implication is that single-cell clones would be required to observe a significant phenotype in particle secretion and the downstream effects of Rab27a or Rab35 KO.

**Figure 2.**
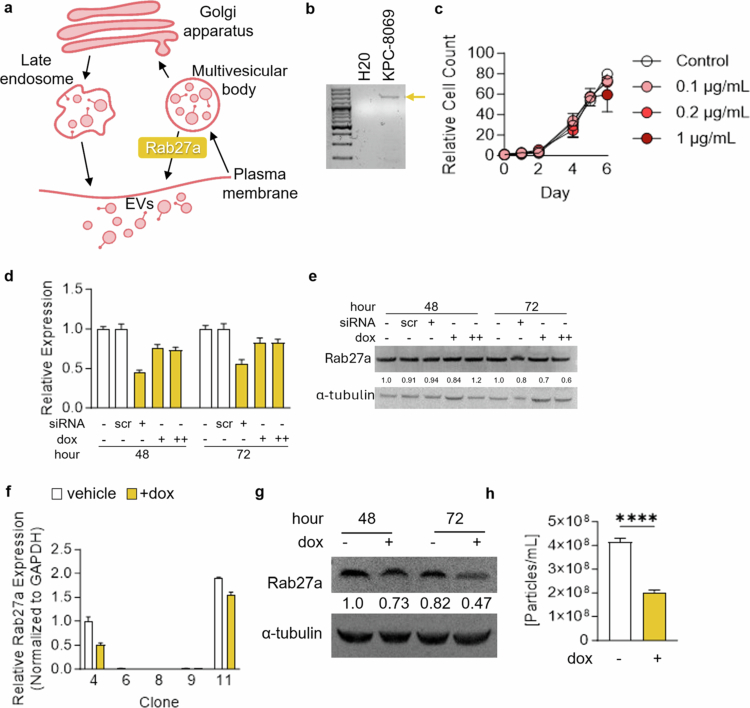
Pooled shRab27a does not KD Rab27a. (a) Schematic of EV production through the action of Rab27a. (b) DNA validation of the full Tet-On shRab27a lentiviral construct following transduction. (c) Growth of KPC cells treated with increasing concentrations of dox. (d) Relative *Rab27a* expression normalized to *Gapdh* in KPC PalmGRET tCD19 shRab27a cells with siRab27a or dox treatment (+ = 0.6 µg/mL, ++ = 3 µg/mL. (e) Immunoblotting of shRab27a cells after 48 and 72 h of siRNA or 3 µg/mL dox treatment. (f) Relative *Rab27a* expression normalized to *Gapdh* in 5 clones with and without 0.3 µg/mL dox treatment. (g) Immunoblotting of shRab27a clone 4 with and without 3 µg/mL dox at 48 and 72 h probed for Rab27a and α-tubulin. (h) Nanoparticle tracking analysis of EVs isolated from shRab27a clone 4 with and without 3 µg/mL dox. *p*-values were calculated using an unpaired two-tailed Student's *t*-test. *p < 0.05; **p < 0.01; ***p < 0.001; ns, not significant.

Next, we also utilized a tetracycline-inducible construct, whereby shRab27a is expressed upon doxycycline (dox) treatment (Figure 2a). This Tet-On system allows for rapid and reversible control of Rab27a expression, which reduces the risk of compensation and makes it advantageous for the KO cell lines we generated. Further, this technique was utilized in an immunocompromised mouse model of PDAC, where Rab27a KD decreased tumor growth.^5^ We transduced KPC-8069 cells to express the shRab27 construct and validated its insertion at the DNA level (Figure 2b). We performed dox sensitivity assays to determine the optimal concentration of dox for our KPC cell line (Figure 2c). Next, we evaluated the efficacy of shRab27a KD with dox treatment (0.6 and 3 µg/mL) compared to Rab27a siRNA treatment for 48 and 72 h (Figure 2d–e). We observed that siRab27a treatment was much more effective than shRab27a at reducing Rab27a expression at the RNA level; however, there were minimal reductions in total Rab27a protein abundance. Based on these findings, we postulated that generating single-cell clones may improve Rab27a KD. We generated single-cell clones and found that of the 11 clones, clone 4 was the only one with significant mRNA Rab27a KD upon 3 µg/mL dox treatment (Figure 2f). After four consecutive days of dox treatment, the Rab27a protein abundance also decreased (Figure 2g). Importantly, we found that these cells also significantly decreased EV secretion, as quantified by nanoparticle tracking analysis (Figure 2h).

**Figure 3.**
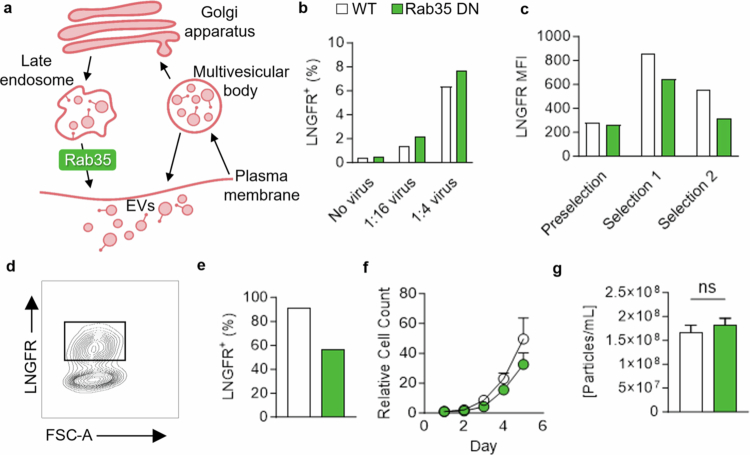
Rab35 DN does not impact EV secretion in KPC cells. (a) Schematic of EV secretion involving Rab35. (b) Lentiviral transduction efficacy of WT (white) vs. Rab35 DN (green) assessed by flow cytometry for LNGFR with 1:16 and 1:4 dilutions of lentivirus. (c) Intensity of LNGFR with bead selection for LNGFR^+^ cells following transduction and two rounds of bead selection. (d) FACS plots of KPC cells transduced with WT or Rab35 DN lentivirus gated for LNGFR^+^. (e) LNGFR^+^ following FACS. (f) Growth of transduced WT vs. Rab35 DN cells. (g) Particle secretion of WT vs. Rab35 DN cells. *p*-values were calculated using an unpaired two-tailed Student's *t* test. *p < 0.05; **p < 0.01; ***p < 0.001; ns, not significant.

We next explored whether engineering our KPC cells to express the dominant negative (DN) mutant of Rab35 S22N would inhibit EV secretion without inducing compensatory mechanisms that may have occurred in our Rab35 KO cells.^8^ We transduced KPC cells with lentivirus to control Rab35 or Rab35 DN (Figure 3a). We ensured that the initial transduction did not exceed 20% of the cells to provide one or fewer insertions per cell. We validated this by staining and performing flow cytometry for LNGFR, which is part of the construct inserted (Figure 3b). We enriched our population by utilizing an LNGFR bead pull-down column and established 60–90% purity (Figure 3c). To increase the purity of our population, we attempted a second bead isolation, which did not improve our purity. Thus, we performed fluorescence-activated cell sorting (FACS, Figure 3d). However, we found no significant difference in cell growth or EV secretion between WT and Rab35 DN cells in the FACS-sorted populations (Figure 3f–g).

**Figure 4.**
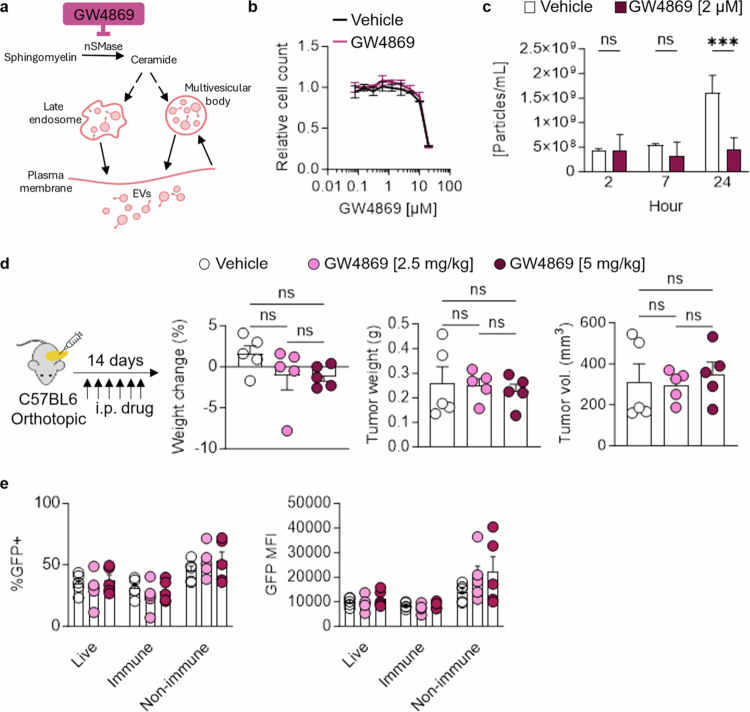
GW4869 treatment does not alter KPC tumor growth. (a) Schematic of the mechanism of action for small molecule inhibitor GW4869 targeting nSMase, limiting the production of ceramide decreasing downstream EV production. (b) Kill curve of KPC PalmGRET cells treated with vehicle or GW4869. (c) Particle secretion time course with vehicle or 2 µM GW4869. (d) KPC PalmGRET cells were orthotopically implanted into the pancreas of C57BL6 mice and treated i.p. with vehicle or GW4869 (2.5 or 5 mg/kg) and euthanized after 14 d. Percent weight change in the mice and final tumor weight and volume. (e) EV import monitored by flow cytometry for the percentage of GFP^+^ and GFP mean fluorescence intensity (MFI) in live, immune (CD45^+^), and nonimmune (CD45^−^) cells. *p-v*alues were calculated using an ordinary one-way ANOVA. *p < 0.05; **p < 0.01; ***p < 0.001; ns, not significant.

As an alternative approach to genetically engineering KPC cells, we tested the small molecule inhibitor of nSMase, GW4869, a key enzyme in the production of ceramides that are required upstream of EV production (Figure 4a). We validated that GW4869 does not significantly kill KPC cells compared to vehicle DMSO alone (Figure 4b). Next, we found that EV secretion significantly decreases with 2 µM GW4869 treatment for 24 h (Figure 4c). We tested the efficacy of GW4869 *in vivo* by orthotopically implanting KPC cells expressing the EV reporter PalmGRET into the pancreas of C57BL6 mice (Figure 4d). After 14 d, we harvested and dissociated the tumors for flow cytometry. We tested two dosing strategies on these mice, which were treated intraperitoneally (i.p.) daily from days 3–13 with vehicle, 2.5 mg/kg, or 5 mg/kg GW4869. We found that mice tolerated both drug doses, although tumor weight and volume were not altered (Figure 4d).

We wanted to assess whether the drug treatment effectively inhibited EV secretion, so we dissociated the tumors and performed flow cytometry. We stained tumors for CD45 to measure EV import (GFP signal) in immune (CD45^+^) and nonimmune (CD45^−^) stromal cells (Figure 4e). We found no change in GFP positivity by percentage or intensity in live, immune, or nonimmune stromal cells. These data suggest that GW4869 treatment did not measurably reduce EV-mediated transfer *in vivo* under the conditions tested, although this conclusion is based on an indirect readout rather than direct quantification of secreted EVs. Future work could evaluate whether there is a decrease in GFP^+^ particles in the interstitial fluid of PDAC tumors with GW4869 treatment to validate whether EV secretion is impaired.

## Discussion

Inhibiting EV secretion has been reported to decrease tumor growth and/or metastasis in mouse models of PDAC.^4^^,^^5^ Here, we leveraged genetic approaches to KD or KO Rab27a or Rab35 in mouse PDAC cells. This approach did not work as anticipated, as one of eleven shRab27a clones had significant KD of EV secretion *in vitro*. Together, these data suggest that our KPC PDAC cell line does not require Rab27a or Rab35 for EV secretion. Importantly, several technical improvements could be made to these studies moving forward. For the generated KO cell lines, we performed all *in vitro* and *in vivo* studies with a pooled cell line that still had a small percentage of WT cells. Although immunoblotting confirmed the KO of Rab27 and Rab35, we cannot rule out the possibility that EV secretion was not inhibited because some WT cells remained. Thus, generating single-cell clones could improve this model. An alternative explanation for why this model does not perform as previously published is the inherent differences between the KPC-8069 cells and the KPC cells used by others.^4^^,^^5^ We aimed to compare the baseline expression of Rab27a to test this and found that, at least relative to the human BxPC-3 PDAC cell line, KPC-8069 cells express very low levels of Rab27a (data not shown). Further, KO of Rab27a in our KPC-8069 cells did not lead to a significant shift in Rab27a expression, suggesting that these cells may rely on other GTPases for EV secretion.

While directly inhibiting EV secretion from tumor cells is mechanistically appealing, small-molecule inhibitors such as GW4869 act broadly and also affect secretion from nontumor cells. We leveraged GW4869 to block ceramide-dependent EV biogenesis. Dr. Sonia Melo and colleagues utilized similar approaches due to the justification that tumor cells secrete high levels of EVs.^4^ However, in our hands, immortalized normal pancreatic ductal cells (HPNEs) release EVs at levels comparable to those in PDAC cell lines (PANC-1, MIA PaCa-2) *in vitro*, suggesting that systemic inhibitors may not be tumor-selective. These findings may explain why GW4869 impaired EV secretion *in vitro*, while we observed no differences *in vivo* as measured by the GFP signal across cell types in the TME. Importantly, our readout relied on EV uptake rather than direct quantification of secreted vesicles from plasma or tumor interstitial fluid, which would provide a more definitive measure of EV inhibition. Further, although we selected doses (5 mg/kg i.p. daily) used in tumor models, effective regimens vary widely in the literature, with some studies reporting up to 50 mg/kg i.p. twice weekly.^14^ Thus, the lack of measurable effects *in vivo* may reflect pharmacokinetic limitations rather than the absence of EV biological activity. Given the complexities of EV signaling and the systemic impact of pan-EV inhibitors, targeting ceramide production has inherent limitations. Future work should explore alternative small molecules, such as spiroepoxide or manumycin A, that may offer improved pharmacological properties for *in vivo* applications.^15^^,^^16^

Overall, these data highlight the challenges and importance of targeted EV secretion from cells. Cells are dynamic and have continuous movement of lipid bilayers, so stopping this movement and the secretion of particles is likely challenging. We have shown that some of the most published techniques do not perform as anticipated in all cells in our laboratory, thus a high-throughput technique to identify key proteins that mediate EV release across diverse biological contexts and disease states is needed. Dr. Kunitake and colleagues published one such approach, leveraging CRISPR-assisted individually barcoded small EV-based release regulator (CIBER) to identify a broad range of genes involved in EV release.^17^ Still, it is essential to approach EV release inhibition cautiously, as EV secretion inhibition should be validated both *in vitro* and *in vivo*. This becomes more challenging in mouse models; however, our group has collected interstitial fluid from our tumors to perform downstream particle flow cytometry to assess whether EV secretion from PalmGRET PDAC cells is inhibited. Future work in the EV space will require rigorous validation of EV secretion inhibition and/or novel strategies to inhibit EV secretion to tease apart the mechanisms by which tumor cells signal to the tumor microenvironment via EVs.

## Supplementary Material

Supplementary material Supplementary Figure 1S: Validation of EV isolation. (a) Protein concentration measured by the absorbance at 280 nm (A280) of SEC fractions 1–14. (b) Immunoblotting of cell lysates (CL) and SEC fractions 3 (pre-EV), 7–10 (EV), and 13–14 (post-EV) from KPC-8069 cells. Blot probed for total protein (Ponceau), EV markers (CD81, TSG101, and ALIX), and a cell lysate control (cytochrome c). (c) Fluorescent nanoparticle tracking analysis of SEC fractions 7–10. (d) Transmission electron microscopy of SEC fractions 3 (pre-EV), 7–10 (EV), and 13–14 (post-EV) at 30,000x (black scale bar = 550 nm) and 150,000× (white scale bar = 50 nm).

Supplementary material Supplementary Figure 1S: EV isolation validation. (a) Protein concentration measured by the absorbance at 280 nm (A280) of SEC fractions 1–14. (b) Immunoblot of cell lysates (CL) and SEC fractions 3 (pre-EV), 7–10 (EV), and 13–14 (post-EV) from KPC-8069 cells. Blot probed for total protein (Ponceau), EV markers (CD81, TSG101, and ALIX), and cell lysate control (cytochrome c). (c) Fluorescent nanoparticle tracking analysis of SEC fractions 7–10. (d) Transmission electron microscopy of SEC fractions 3 (pre-EV), 7–10 (EV), and 13–14 (post-EV) at 30,000x (black scale bar = 550 nm) and 150,000x (white scale bar = 50 nm).

## Data Availability

The data that support the findings of this study are available from the corresponding author upon reasonable request.
