# Supplementary material for: Challenges and caveats in manipulating extracellular vesicle secretion from pancreatic cancer cells
Source: Cancer Biol Ther. 2025 Oct 25;26(1):2569946. doi: 10.1080/15384047.2025.2569946 (PMC12562733; doi:10.1080/15384047.2025.2569946)
Supplement: Supplementary material — Supplementary Figure 1S: Validation of EV isolation. (a) Protein concentration measured by the absorbance at 280 nm (A280) of SEC fractions 1–14. (b) Immunoblotting of cell lysates (CL) and SEC fractions 3 (pre-EV), 7–10 (EV), and 13–14 (post-EV) from KPC-8069 cells. Blot probed for total protein (Ponceau), EV markers (CD81, TSG101, and ALIX), and a cell lysate control (cytochrome c). (c) Fluorescent nanoparticle tracking analysis of SEC fractions 7–10. (d) Transmission electron microscopy of SEC fractions 3 (pre-EV), 7–10 (EV), and 13–14 (post-EV) at 30,000x (black scale bar = 550 nm) and 150,000× (white scale bar = 50 nm). [file KCBT_A_2569946_SM9676.docx]

**Supplementary Figure 1S: EV isolation validation.**

**(a)** Protein concentration measured by the absorbance at 280 nm (A280) of SEC fractions 1-14. **(b)** Immunoblot of cell lysates (CL) and SEC fractions 3 (pre-EV), 7–10 (EV), and 13–14 (post-EV) from KPC-8069 cells. Blot probed for total protein (Ponceau), EV markers (CD81, TSG101, and ALIX), and cell lysate control (cytochrome c). **(c)** Fluorescent nanoparticle tracking analysis of SEC fractions 7-10. **(d)** Transmission electron microscopy of SEC fractions 3 (pre-EV), 7-10 (EV), and 13-14 (post-EV) at 30,000x (black scale bar = 550 nm) and 150,000x (white scale bar = 50 nm).
