# Supplementary material for: Challenges and caveats in manipulating extracellular vesicle secretion from pancreatic cancer cells
Source: Cancer Biol Ther. 2025 Oct 25;26(1):2569946. doi: 10.1080/15384047.2025.2569946 (PMC12562733; doi:10.1080/15384047.2025.2569946)
Supplement: Supplementary material — Supplementary Figure 1S: EV isolation validation. (a) Protein concentration measured by the absorbance at 280 nm (A280) of SEC fractions 1–14. (b) Immunoblot of cell lysates (CL) and SEC fractions 3 (pre-EV), 7–10 (EV), and 13–14 (post-EV) from KPC-8069 cells. Blot probed for total protein (Ponceau), EV markers (CD81, TSG101, and ALIX), and cell lysate control (cytochrome c). (c) Fluorescent nanoparticle tracking analysis of SEC fractions 7–10. (d) Transmission electron microscopy of SEC fractions 3 (pre-EV), 7–10 (EV), and 13–14 (post-EV) at 30,000x (black scale bar = 550 nm) and 150,000x (white scale bar = 50 nm). [file KCBT_A_2569946_SM9669.pdf]

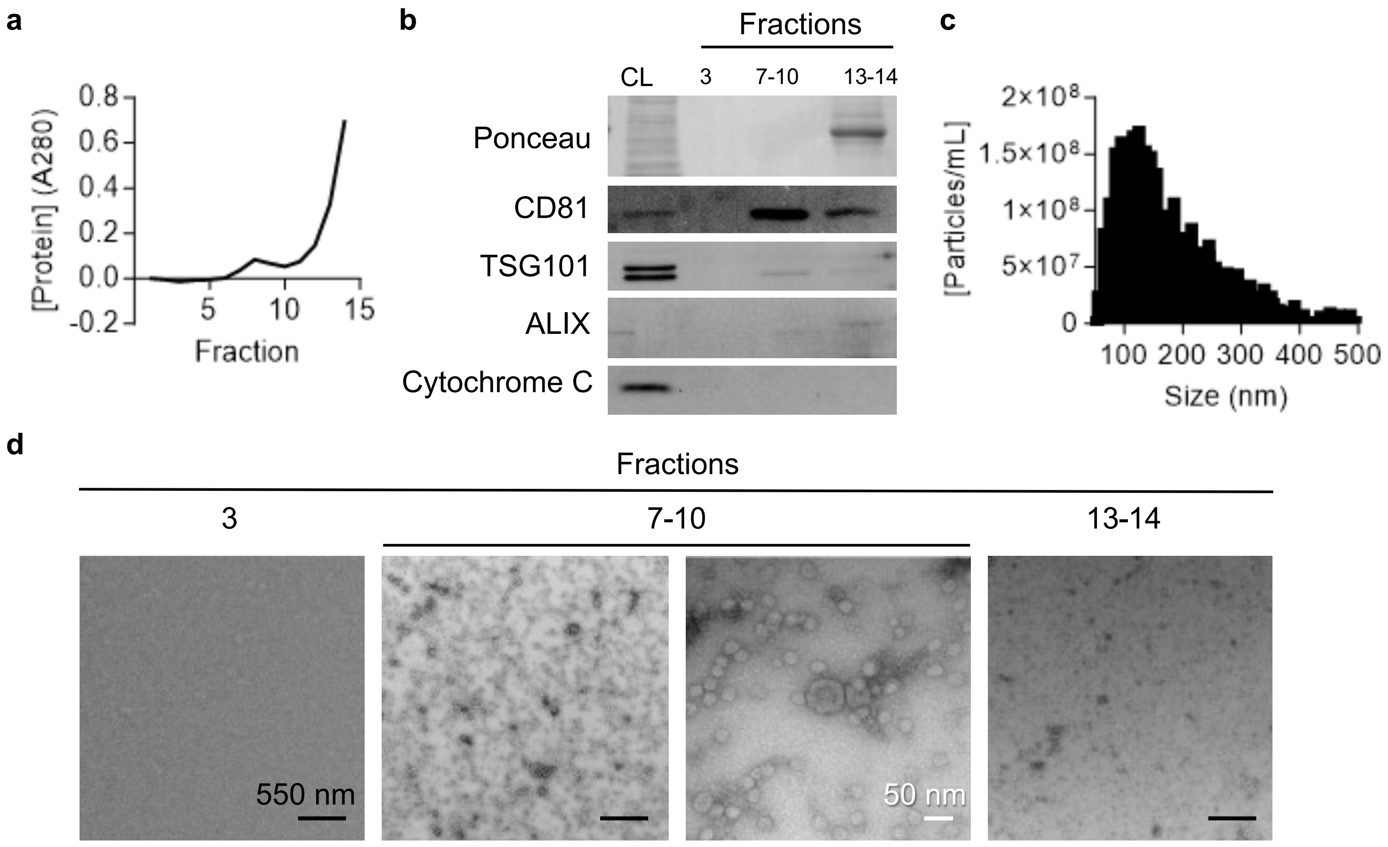

Supplementary Figure 1S: EV isolation validation.

(a) Protein concentration measured by the absorbance at 280 nm (A280) of SEC fractions 1-14. **(b)** Immunoblot of cell lysates (CL) and SEC fractions 3 (pre-EV), 7–10 (EV), and 13–14 (post-EV) from KPC-8069 cells. Blot probed for total protein (Ponceau), EV markers (CD81, TSG101, and ALIX), and cell lysate control (cytochrome c). **(c)** Fluorescent nanoparticle tracking analysis of SEC fractions 7-10. **(d)** Transmission electron microscopy of SEC fractions 3 (pre-EV), 7-10 (EV), and 13-14 (post-EV) at 30,000x (black scale bar = 550 nm) and 150,000x (white scale bar = 50 nm).
